# Network science and explainable AI-based life cycle management of sustainability models

**DOI:** 10.1371/journal.pone.0300531

**Published:** 2024-06-13

**Authors:** Ádám Ipkovich, Tímea Czvetkó, Lilibeth A. Acosta, Sanga Lee, Innocent Nzimenyera, Viktor Sebestyén, János Abonyi

**Affiliations:** 1 HUN-REN-PE Complex Systems Monitoring Research Group, University of Pannonia, Veszprém, Hungary; 2 Climate Action and Inclusive Development (CAID) Unit, Global Green Growth Institute, Jung-gu, Seoul, Republic of Korea; 3 Sustainability Solutions Research Lab, Faculty of Engineering, University of Pannonia, Veszprém, Hungary; Abdul Wali Khan University Mardan, PAKISTAN

## Abstract

Model-based assessment of the potential impacts of variables on the Sustainable Development Goals (SDGs) can bring great additional information about possible policy intervention points. In the context of sustainability planning, machine learning techniques can provide data-driven solutions throughout the modeling life cycle. In a changing environment, existing models must be continuously reviewed and developed for effective decision support. Thus, we propose to use the Machine Learning Operations (MLOps) life cycle framework. A novel approach for model identification and development is introduced, which involves utilizing the Shapley value to determine the individual direct and indirect contributions of each variable towards the output, as well as network analysis to identify key drivers and support the identification and validation of possible policy intervention points. The applicability of the methods is demonstrated through a case study of the Hungarian water model developed by the Global Green Growth Institute. Based on the model exploration of the case of water efficiency and water stress (in the examined period for the SDG 6.4.1 & 6.4.2) SDG indicators, water reuse and water circularity offer a more effective intervention option than pricing and the use of internal or external renewable water resources.

## Introduction

To achieve Sustainable Development Goals (SDGs) and their targets, regular assessments are needed to track the progress of countries and identify areas where more effort is necessary [1]. These assessments may require a range of data and information, including indicators on social, economic, and environmental issues [2]. Evaluations can also involve consultation with various stakeholders, including governments, civil society organizations, and the private sector [3]. The main objective is to provide a complete picture of progress toward the SDGs, identify challenges and opportunities [4], and inform policies and actions that can help accelerate progress [1].

The complex relationships between policies and social, economic, and environmental issues require models [5] and analyses that consider each issues and predict the performance of sustainable development in countries [6]. Model-based assessment of sustainability planning provides decision-makers with a powerful tool to understand the complex interdependencies between social, economic, and environmental systems and design policies and strategies that are likely to lead to more sustainable outcomes. It involves developing quantitative structural equation models that represent the systems that are being considered and using these models to explore different scenarios and assess the likely impacts of different policy choices. In assessing aggregated SDG indicators, effective decision support requires models that are suitable for identifying potential intervention points and for establishing knowledge in political strategy creation.

As SDG indicators are aggregated, effective decision support requires models that are suitable to identify potential intervention points and establish knowledge in the creation of political strategies. Country-specific factors such as unique development phases, policies, and databases can make it difficult to develop accurate and reliable system dynamics models. Therefore, it is important to pay attention to the life cycle of models and the difficulties that may arise during each stage, such as initial contextualization, data utilization, model creation, analysis, implementation, and monitoring.

A common challenge in developing reliable complex systems is model identification to accurately represent the workings of the system. Regarding sustainability, this challenge can arise due to the detailed structure of SDGs, data and information limitations, and modeling techniques. The development of structured models requires broad experience in modeling techniques and validation methods and relies heavily on data and information. An additional requirement is that the relationships between variables, and their change in behavior over time must be known [7]. In this regard, life cycle-based assessment encompasses the various stages of model development, including initial contextualization and data utilization for design, the creation of a complex model, the derivation of analysis, the implementation of the model, and the monitoring and revision of the model to improve its accuracy.

The primary objective of this paper is to highlight approaches that support structural equation model-based assessment of sustainability planning, identify the contribution of variables to the SDG indicators based on historical data, and promote evidence-based policy development. We propose methods based on network and data analysis to support modeler work and obtain more accurate, automatic, and efficient model development procedures during the entire life cycle of models. We introduce the potential for Shapley value utilization to identify the contribution of each variable in the model to the output SDG indicators. Furthermore, we highlight the opportunities for life cycle-based modeling as well as the links between life cycle phases with the help of network and data science technologies. It is important to highlight that we do not mean the identification of environmental impacts by the expression ‘life cycle’, we consider the modeling process as a life cycle, which draws attention to the fact that during the development of expert systems (in their life cycle) different steps can be followed to develop the concept, for which the tools proposed in this research can be used.

Structural equation models can be represented as networks, and sustainable development-related problems can be evaluated using network science tools [8]. Network analysis can be useful in understanding the complex networks of stakeholders, institutions, and processes that influence sustainability outcomes. Furthermore, it can be used to understand the relationships between different SDG goals and targets. For example, network tools can help identify the interdependencies between different goals and understand how progress in one area can impact others [9]. The important variables of the model can be identified that can serve as possible intervention points. Additionally, data-based methods can be used to support the continuous development and analysis of structural equation models to assess progress toward the SDGs. Data-driven methods can reveal the relationship between different variables, support understanding of the underlying mechanisms that drive these relationships, identify patterns and trends, and the potential impact of different interventions on the system [10].

The related models are usually static in a way that the developed models are not further improved, validated, and maintained throughout the modeling life-cycle. The modeling structure is usually not prepared to integrate and track changes, whereas we are and will be witnessing more and more dramatic changes because of the radical steps taken in the direction of polycentrism and green transformation. Therefore, there is an emerging need for the model life-cycle-based development.

By combining network and data analysis throughout the entire model life cycle, stakeholders can gain a more complete understanding of the complex interactions and relationships that influence the achievement of the SDGs. This life cycle-based assessment of models can be used to ensure that models are developed, deployed, and maintained in a reliable, efficient, and effective manner. In this study, we consider the intertwining of MLOps and CRISP-ML (Q) (Cross-Industry Standard Process for the development of Machine Learning applications with Quality assurance methodology) life cycles. MLOps is an end-to-end framework of the machine learning (ML) development process and supports automation principles and includes three phases: design, model development, and operation [11]. This framework is highly intertwined with CRISP-ML(Q), which consists of six iterative phases with varying sequences: business and data understanding, data preparation, modeling, evaluation, deployment and monitoring, and maintenance [12].

The system model of the Sustainable Development Goals can take into account changes over time in the relationships between the SDG goals and the targets [13], so there is a significant need to maintain the models, as the behaviour of the system is dependent on time and maturity with a special focus on responses to political challenges [14], including critical and success factors in economic sectors and national infrastructures [15]. Action-focused approaches increase their utility in decision-making, so the scenario derivation supports the formulations of reasonable actions [16].

Additional variables could be added to the explanation of ‘unexplained’ interactions in the SDG models [13], which can be supported by machine learning [15]. Modeling efforts can be exploited with a feedback-rich structure of models [17].

Therefore, the potential contributions and the structure of this article are as follows:

- The life cycle-based model development and possible application of data and network science tools in each life cycle phase are discussed in the *Sustainability focused model life cycle management section*.
- *The methodology of using network and data science tools in model identification and development is explored in the Development of models based on network science and explainable AI tools section. This section emphasizes the challenges inherent in model development and how the utilization of network analysis and the Shapley value can mitigate these difficulties. They serve as sensitivity analyses by promoting the understanding of which variables have the greatest direct or indirect impact on changes in SDG indicators through the model network based on historical data*.
- *A case study is presented in the Application of network and data analysis for model development section to demonstrate the effectiveness of the proposed methods. The case study is conducted on a submodel developed by the Global Green Growth Institution for Hungary*.

It is imperative to underscore that the evaluation of models is contingent upon the data they reflect, meaning that the results are primarily influenced by the data. Consequently, insufficient quality of the data can lead to incorrect inferences about the model’s quality and the effects of variables. Furthermore, we must consider that different economies or environmental backgrounds may exhibit different relationships. Therefore, a homogeneous sample is essential, requiring a clear definition of the modeled area. Naturally, the structure of models can also be flawed, and identifying structural errors may not be straightforward. MLOps primarily focuses on retraining the same model to address this issue. However, the methodology enables to creation of a new model by incorporating new variables and insights.

Nevertheless, we believe that the network and data analysis approach to model-based sustainability planning offers valuable information throughout the entire model life cycle. Furthermore, by identifying the direct and indirect contributions of variables to changes in the SDGs, this data-driven approach can serve as a fundamental basis for evaluating the efficacy of policy intervention. The relevancy of the proposed method is proved and validated through the Global Green Growth Institute (GGGI) who applied the method in real-life sustainability models. The interpreted application study relies on a real-life example that is developed by the Global Green Growth Institute, which is a treaty-based international, inter-governmental organization dedicated to supporting and promoting strong, inclusive, and sustainable economic growth in developing countries and emerging economies. The developed method will be an integrated tool that will be used for their work, enabling policymakers and researchers to assess the environmental and economic impacts of various policy interventions, and fostering informed decision-making for sustainable development initiatives worldwide.

## Materials and methods

### Sustainability focused model life cycle management

Environmental systems exhibit a high degree of complexity and dynamics, characterized by diverse interacting variables that may vary over time. Models that aim to capture such complexities are often susceptible to uncertainties attributable to the evolving nature of environmental systems, the lack of data, and the adaptability of the model to local conditions. The localization of model structures necessitates the customization of generalized models to reflect unique characteristics of countries, regions, or cities, including but not limited to social, economic, and environmental developmental levels, as well as diverse policy goals and interventions aimed at enhancing sustainable development.

The systematic development of these models can be supported with machine learning techniques to incorporate data-driven insights and evidence-based policy assessment. MLOps and CRISP-ML are systematic frameworks that consider the whole life cycle of data-driven models from design and business understanding until the operation phase and model maintenance [18]. This life cycle-based model evaluation can be used to ensure that models are developed, deployed, and maintained in a reliable, efficient, and effective manner. These concepts are fundamentally iterative and exploratory, so depending on the results from the later phases, the reexamination of earlier steps may be needed.

**Fig 1:**
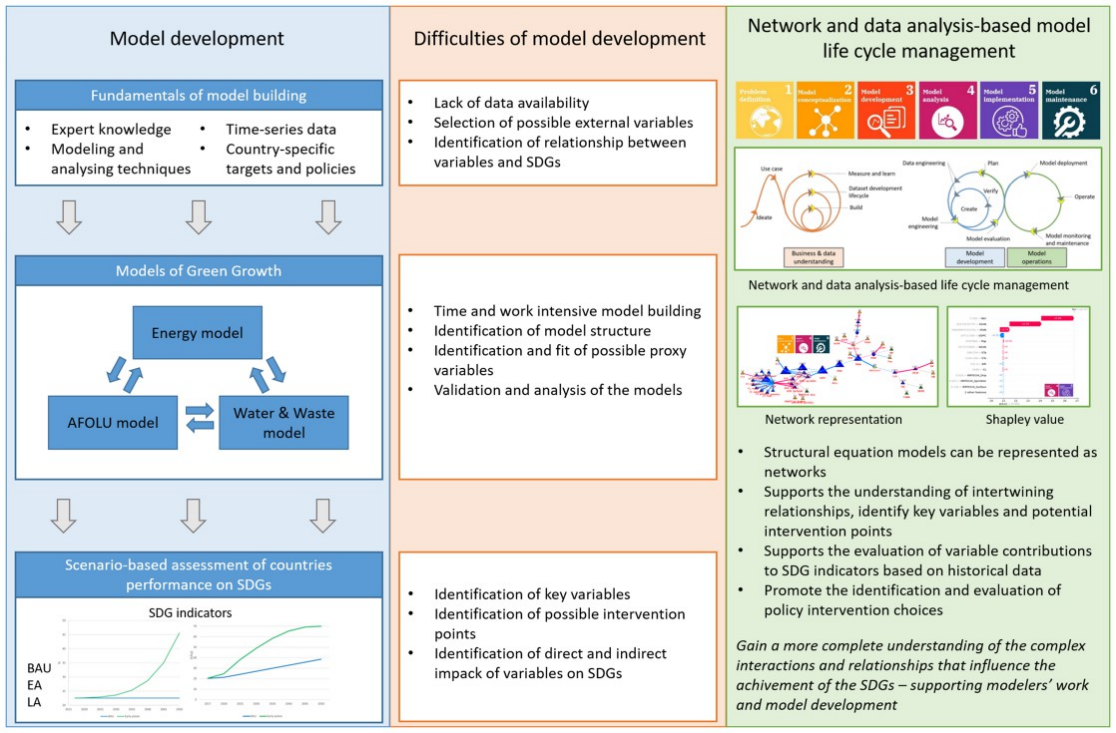
The framework of applying network and data analysis-based life cycle management of sustainability model development.

In Fig 1, the framework for applying data and network analysis-based life cycle management for the development of sustainability models is shown. The blue block represents model development, where model building is based on expert knowledge and time series data and requires the use of modeling and analyzing techniques, as well as country-specific targets and policy implications for accuracy. The Global Green Growth Institute built models for assessing and predicting green growth by linking the energy, agriculture, forestry and other land use (AFOLU), and water and waste models [19]. The orange block indicates the difficulties of model development as mentioned above, such as lack of data availability, identification of possible external variables and their relationship, model validation, assessment of intervention points, analysis of the effect of variables on SDGs, *etc*. The green block represents a model life cycle-based solution for model development utilizing the tools of network and data sciences.

By following the CRISP-ML methodology, organizations can ensure that models are developed, evaluated, and deployed in a transparent and structured manner, which can help to improve decision making and support long-term planning and management of environmental systems. Additionally, the CRISP-ML provides a clear roadmap for monitoring and maintaining the model, allowing continuous improvement and adaptation to new information and scenarios.

**Fig 2:**
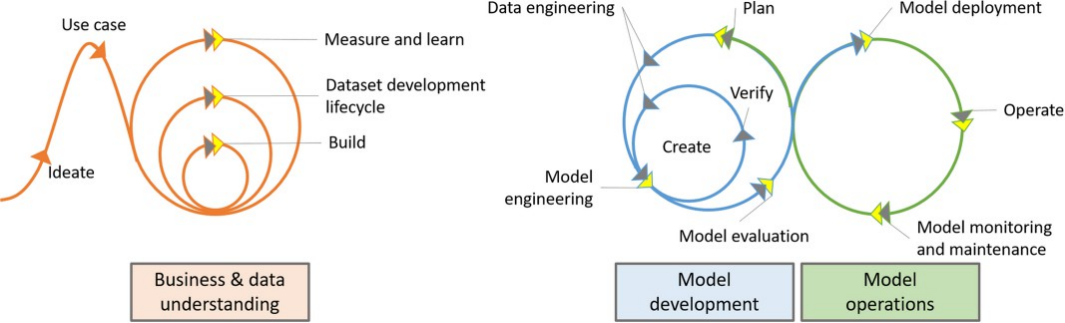
Schematic representation of the connections between MLOps and CRISP-ML (based on [18]). The line colors represent the different stages (orange—business and data understanding, blue—model development, green—model operation) and the arrow colors identify where network and data science tools can be applied (network science—grey, data science—yellow).

The schematic workflow and connections between MLOps and CRISP-ML are illustrated in Fig 2, where the line colors represent the different stages (orange—business and data understanding, blue—model development, green—model operation) and the arrow colors identify where network and data science tools can be applied (network science—grey, data science—yellow).

In Table 1, the intersection of MLOps, CRISP-ML life cycle, and the potential applicability and implementation of network and data science techniques are examined within the framework of model-based assessment of Sustainable Development Goals.

**Table 1:** Data and network science methods supporting sustainability model development in the MLOps and standard modeling life cycle.

MLOps	CRISP-ML dimensions	Model relevance	Data science relevance	Data science methods	Network science relevance	SDG relevance	References
Design	Business understanding	S1 Fig Problem definition	Understand the problem, goals and objectives of the project, by analyzing historical data, identifying trends, and patterns and understanding the relationships between different variables.	Exploratory Data Analysis (EDA) [20], Descriptive statistics, Data mining [21], Data visualization, Causality analysis [24]	Identify the key variables and relationships that are important for understanding the behavior of the system, by creating a conceptual model of the system.	Identify the interlinkages between SDG goals [25], targets [36] and indicators [9]. Identify the connection between model elements and SDGs. Identify dependencies among risks associated with SDGs [26].	[9, 20, 21, 23–27, 36–42]
	Data understanding	S2 Fig Model conceptualization	Collecting and verifying the data quality regarding the structured issues and, finally deciding upon whether the project should be committed.	Data cleaning, Data exploration [20], Correlation analysis [27], Causality analysis [23], Data visualization, Shapley value [42]			
Model development	Data preparation	S3 Fig Model development	Representing sustainability-related concepts and structured knowledge of the relationship between model elements. Producing a data set for the modeling design phase by gathering and linking data from diverse data sources.	Feature engineering, Data imputation, Feature scaling, Data normalization, Data selection, Data cleaning, Ontology modeling, Knowledge graphs, Open linked data	Defining knowledge graphs representing the relationship between model elements. Gather and analyze the data that is needed to validate the model, by identifying patterns and structures in the data that can be used to inform the development of the model.	Represent the SDGs and their interrelationships with other ontologies to facilitate the integration and analysis of data applying to the SDGs. Provides transparent and reproducible analysis, establishing a shared vocabulary and framework for the description and sharing of data and models associated with the SDGs.	[43–48]
	Modeling		Creating models that satisfy the given constraints and requirements.	Regression analysis, Time series analysis [28], Clustering, Decision trees, Random forest, Neural networks [32], Bayesian methods [49], and other machine learning algorithms [29], Digital Twins [35], Composite indicators Cross-validation, Hyperparameter tuning, Sensitivity analysis, Classification, Clustering, Regression analysis, Frequent itemset/pattern mining, Visualization	Build and test the model, by using network metrics such as centrality to identify key elements in the system and to understand the overall connectivity of the system.	Predictions [50], simulations, structural model development, risk assessment [26]	[26, 28–35, 49–52]
	Evaluation	S4 Fig Model analysis	Design and analysis are intertwined and as new change options are proposed, they are analyzed using data analysis techniques. The performance, robustness and explainability must be evaluated.		Analyze the results of the simulation and to identify the best course of action for achieving a desired outcome, by identifying patterns and structures in the results that can be used to inform decision-making.	Identify and categorize countries, regions based on their performance on SDGs. Identify patterns and key variables which can be possible intervention points to succeed on SDGs.	[5, 25, 29, 38, 40, 52]
Operations	Deployment	S5 Fig Model implementation	Before rolling out a model to all, it is best practice to deploy it first to a small subset and evaluate its behaviour.	Model deployment, Simulation models, Digital Twins [35], Model interpretability, Monitoring [53], Web page development, Dashboard development, Model maintenance	Identify the most critical elements in the network that need to be targeted for the implementation of policies or strategies that have been identified as the best course of action.	Identify transition trajectories, different scenarios based on diverse strategies. Identify the possible performance on SDGs based on the diverse strategies.	[5, 30, 35, 51, 52]
	Monitoring and Maintenance	S6 Fig Model maintenance	Monitoring and maintenance processes to assure quality performance and identify corrective actions. The model has to adapt to changes in the environment.			Continuous monitoring [54], improvement and validation of the models. Monitor the transition trajectories. Identify possible external variables and mitigation actions for better accuracy.	[53–55]

According to Studer *et al*. [12] the *business and data understanding phase* includes defining objectives, collecting and verifying data quality, and assessing the project. This phase involves identifying the project’s scope, risks, and success criteria such as measurable features, systems alignment, Key Performance Indicators (KPIs), feasibility, and data availability, which are critical components of model development. Regarding data science methods, exploratory data analysis (EDA) provides insight and understanding of databases, visualization of potential relationships between variables, detection of outliers and anomalies, development of simple models (predictive or exploratory), the precondition of data [20]. Data mining and machine learning methods can be valuable tools for discovering knowledge from databases [21]. Integration of data mining and system dynamics supports evidence-based decision-making and a better understanding of the dynamics and complexity of a system [22]. The relationships between, *e.g*., sustainability pillars [23], the interconnectedness of SDGs [24], and their patterns can be identified through causality analysis that is important from a policy point of view. The causal relationships [24] and complex interactions of the SDGs can be mapped through network analysis [25]. For example, a probabilistic network model can be used to explore the dependencies among the risks associated with the SDGs [26].

During the phase of model development, the data is preprocessed and delivered in a format that is suitable for integration with the model. This process, also known as data preparation, includes the selection, cleaning, and standardization of the data. The modeling activity involves selecting appropriate models, incorporating domain knowledge, evaluating, validating, and documenting the models. The data preparation stage is supported by techniques such as feature engineering, scaling, normalization, and data imputation [27]. There are various methods that can be applied to the building and analysis of models, such as time series analysis and forecasting [28], regression analysis, clustering, classification, dimension reduction techniques [29], Monte Carlo simulation [30], system dynamics modeling [31], network analysis [25], neural networks [32], Bayesian networks [33], and composite indicators [34]. These methods can be integrated to provide a more comprehensive understanding of the system under study. It is worth noting that, while these methods provide valuable information, the identification of other factors such as dynamics, uncertainties, and external events contribute to a more complete understanding of the system.

This will be the basis for *model operations*, where the models are continuously deployed, evaluated, and monitored, as well as fine-tuned if necessary. It is considered good practice to first deploy a model to a small subset and evaluate its behavior prior to extensive implementation *e.g*. transition trajectories, and digital twin simulation based on scenarios may help improve understanding of a phenomenon [35].

The literature on utilizing a network and data-driven tools for the assessment of SDGs has been growing, but their integration and contribution throughout the entire model life cycle remains an area of underexplored research, even though they are used in the steps. Developing a framework that systematically evaluates the potential and contribution of network and data science techniques throughout the model life cycle could prove to be a valuable asset in supporting the work of modelers. In this way, the integration of data-driven techniques and network-based analysis of the system can improve the accuracy, robustness, interpretability, and efficiency of environmental system dynamics models, by allowing the selection of the most relevant variables to the problem, and by identifying key drivers and relationships, it helps organizations and decision-makers to better understand the dynamics of environmental systems and make more informed decisions.

### Development of models based on network science and explainable AI tools

The MLOps approach introduces much-needed structure and efficiency to the development of machine learning and network science models. By combining MLOps practices with explainable AI tools, organizations can leverage the power of these models across different domains, ensuring transparency and interpretability, which in turn fosters greater trust in AI-driven decision-making. In this context, we present a novel Shapley value-based method in Section, which complements the MLOps life cycle by evaluating the contributions of variables to the model. This approach promotes a better understanding of the model, as well as the data, and facilitates model conceptualization, development, and analysis. Furthermore, in Section we highlight the significance of combining the Shapley value and network science in identifying key drivers of model behavior.

#### Shapley value-based evaluation of variable contribution

Complex models are often represented in the form of structural equation models (SEMs), whose equations describe the relationship between variables [56]. These structured equation models can be represented and analyzed as networks. Combining the measures of the structural network with the Shapley value [57] has a great potential to achieve a more complete understanding of the system. As the Shapley value defines the contribution of the variables, in this regard, these values can be applied as weights of the network edges. Thus, a deeper understanding of the interactions of the models can be achieved, which can lead to valuable insights for model development and analysis. The combination of Shapley value and networks is emerging, and has been utilized for example to discover influential nodes in social network [58], identify centrality in weighted and unweighted networks [59], identify individuals’ performance in group influence within a real-world social network [60], used as an extension to betweenness centrality and define a new metric called stress centrality [61]. Furthermore, the Shapley value-based interpretation of feature contribution to model predictions have been applied in a variety of studies, such as predicting antifungal peptides [62], anti-tubercular peptides [63], or anti-inflammatory peptides [64].

Consider a set of *V* that contains *n* number of variables $\left[{\hat{x}}_{1}^{\left(t\right)},\ldots ,{\hat{x}}_{l}^{\left(t\right)},x_{l+1}^{\left(t\right)},\ldots ,x_{n}^{\left(t\right)}\right]$ with *l* number of observed input variables ($\hat{x}$) and *n* − *l* number of derived variables. The *kth* structural model predicts the *kth* derived variable $x_{k}^{\left(t\right)}$ at a given time *t* = 1, …, *T*. The model *f*_*k*_ requires the set of inputs $\left(\chi_{k}^{\left(t\right)}\subseteq V\right)$ and parameters *θ*_*k*_ to predict a variable:

$$\begin{array}{c} x_{k}^{\left(t\right)}=f_{k}(\chi_{k}^{\left(t\right)},\theta_{k}) \end{array}$$ (1)

where $x_{k}^{\left(t\right)}$ denotes the predicted variable, *f*_*k*_ defines the structural equation, $\chi_{k}^{\left(t\right)}$ is considered as the set of input variables, while *θ*_*k*_ stands for the parameters of the equation.

A structural equation model is built from several equations; the model structure can be hierarchical, the output of an equation may be realized as an input of another function.

$$\begin{array}{c} \chi_{k}^{\left(t\right)}=\{x_{j}^{\left(t\right)}|a_{j,k}\neq 0\},j=1,\ldots ,n;t=1,\ldots ,T;x_{j}^{\left(t\right)}\in V \end{array}$$ (2)

where $\chi_{k}^{\left(t\right)}$ is the set of variables at time *t* for function *f*_*k*_. The *jth* variable is denoted by $x_{j}^{\left(t\right)}$. Set *V* contains all variables, *a*_*j*,*k*_ stands for the one-way connection between the *jth* input and the *kth* output variable.

Moreover, data may differ by context, so the models must be parameterized properly. In some cases, parameters may require re-identification as time goes on; new technological and cultural changes may trigger a need for adjustment in the model behavior. One possible solution is to minimize a cost function, *e.g*. the mean squared error of the prediction of the model to an observed output.

$$\begin{array}{c} \underset{\theta_{k}}{\text{min}}\frac{1}{T}\sum_{t=1}^{T}(x_{k}^{\left(t\right)}-f_{k}(\chi_{k}^{\left(t\right)},\theta_{k}){)}^{2} \end{array}$$ (3)

The inclusion of extraneous variables is a potential issue that may arise during the process of developing a model. Therefore, it is important to determine which variable may be relevant to the dependent variable, as it may result in a less convoluted and more accurate model. Establishing the contribution of a variable to the model may enable us to filter out extraneous ones, while also determining the highest contributor, known as biases. However, the impact of one variable should include all contributions to the model, including the added value of cooperation with a group. As such, the average marginal contribution is required, which is often determined by the Shapley value [57].

The marginal contribution requires marginalizing over (“averaging out”) the variables that are excluded from the evaluated selected group of variables. Consider $x_{k}^{\left(t\right)}$ as the dependent variable, $\chi_{k}^{\left(t\right)}$ the set of independent variables. If marginal contributions of a group variable are required, then a contribution evaluation function *ϕ*(⋅) is required for a subset. The excluded variables are marginalized over (averaged out), and, therefore, the contribution of the selected variables is returned. The contribution should be understood as a difference from an expected value (total average of the predictions) [65]:

$$\begin{array}{c} \phi (\chi_{k}^{\left(t\right)}/\left\{x_{j}^{\left(t\right)}\right\})=\int f_{k}(\chi_{k}^{\left(t\right)},\theta_{k})\;dx_{j}\;\;-E\left[f_{k}\right]; \end{array}$$ (4)

where $\phi (\chi_{k}^{\left(t\right)}/x_{j}^{\left(t\right)})$ defines the marginal contribution of variable $x_{j}^{\left(t\right)}$. Here, the expected value is approximated as the average of the model predictions: $E\left[f_{k}\right]\approx \frac{1}{T}\sum_{t=1}^{T}f_{k}(\chi_{k}^{\left(t\right)},\theta_{k})$.

The classical way to approach the Shapley value (calculation of the average marginal contribution) is by subtracting the contribution of a subset of variables with and without the selected variable ($x_{j}^{\left(t\right)}$), and taking their weighted average [57], summed over all possible subsets:

$$\begin{array}{c} S_{j,k}^{\left(t\right)}=\frac{1}{|\chi_{k}^{\left(t\right)}|!}\sum_{P\subseteq \chi_{k}^{\left(t\right)}/\{x_{j}^{\left(t\right)}\}}\phi (P\cup \{x_{j}^{\left(t\right)}\})-\phi (P) \end{array}$$ (5)

where *S*_*j*,*k*_ denotes the Shapley value, *P* denotes a subset of variables $\chi_{k}^{\left(t\right)}$. *ϕ*(*P*) denotes the marginal contribution of the set without, $\phi (P\cup x_{j}^{\left(t\right)})$ defines the marginal contribution of the subset with the examined variable.

As the Shapley value is computationally intensive, it is often approximated by Monte Carlo simulation. For a set with *n* variables, if all possible subset orders are considered, the operation time would be factorial (*O*(*n*)!). As such, variable subsets are randomly sampled with a *M* number of permutations rather than calculating the contribution of each subset. The calculation of the Monte Carlo-based Shapley value requires a set for averaging out the combination of the variables (*e.g*. substituting the expected value of the variable to the set), and the other is for the original values. As such, the computational time can be reduced to *O*(*nM*). The formalization of the approximation of the contribution of the variable can be considered as [66]:

$$\begin{array}{lll} {\hat{\phi }}_{j}({\hat{P}}_{m}) & = & f_{k}({\chi_{k}^{\left(t\right)}}_{\left[xi=E(x_{i}^{\left(t\right)})\right]},\theta_{k})-f_{k}({\chi_{k}^{\left(t\right)}}_{\left[x_{i}=E(x_{i}^{\left(t\right)}),x_{j}^{\left(t\right)}=E(x_{j}^{\left(t\right)})\right]},\theta_{k}),\\ i,j & \notin & m,i\neq j\\ {\bar{S}}_{j,k}^{\left(t\right)} & = & \frac{1}{\left|M\right|}\sum_{m\in M}{\hat{\phi }}_{j}({\hat{P}}_{m}) \end{array}$$ (6)

where *M* is a population of a randomly sampled set of feature combinations, *m* is a feature combination in *M* with variables that are not marginalized, ${\bar{S}}_{j,k}^{\left(t\right)}$ denotes the approximation of the Shapley value $S_{j,k}^{\left(t\right)}$. _$\left[x_{i}=E\left(x_{i}^{\left(t\right)}\right)\right]$_ suffix defines the set of variables, where the specific variables (here, the *ith*) is fixed to an expected value.

The Shapley value aims to explain the change in the value function compared to the expected value, therefore, the sum of the Shapley values for a model must return the difference between the function value at point *t* and the expected value:

$$\begin{array}{c} \sum {\bar{S}}_{j,k}^{\left(t\right)}=f_{k}\left(\chi_{k}^{\left(t\right)},\theta_{k}\right)-E\left[f_{k}\right] \end{array}$$ (7)

Eq 7 defines the efficiency property of the Shapley value, which is to be used to scale individual values between [0, 1]. The marginal contribution of a variable can be defined for an individual sample (*e.g*. contributions of variables for year 2000), or for each to provide information of the trends over the year. For the *jth* input of the *kth* model, the individual and mean Shapley is as follows:

$$\begin{array}{c} w_{j,k}=\{\begin{array}{cc} \frac{{\bar{S}}_{j,k}^{\left(t\right)}}{\sum_{i\in \chi_{k}^{\left(t\right)}}\left|{\bar{S}}_{i,k}^{\left(t\right)}\right|} & \text{if}\;d_{ind}=1\\ \frac{\frac{1}{T}\sum_{t=1}^{T}{\bar{S}}_{j,k}^{\left(t\right)}}{\sum_{i\in \chi_{k}^{\left(t\right)}}\frac{1}{T}\sum_{t=1}^{T}\left|{\bar{S}}_{i,k}^{\left(t\right)}\right|} & \text{if}\;d_{ind}=0 \end{array} \end{array}$$ (8)

where *d*_*ind*_ defines a dummy variable as to whether the weight should be the individual or the average Shapley value. The first member defines the normalized individual contribution, while the second denotes the normalized mean contribution. For a proper weight matrix (**W**), all *d*_*ind*_ should take the same value. If the weight type is selected, then for each model, a vector can be defined where relevant inputs to the models have contributions, *i.e*. the Shapley value is designated as a zero for diagonals (so that there is no cycle) and observed inputs, who have no models.

As shown above, the Shapley value provides a perfect measure to establish the direct connection between variables, making it particularly valuable in the context of MLOps. In the development and deployment of hierarchical models within the MLOps framework, the Shapley value can be used to assess the individual contributions of variables, aiding in the interpretation, monitoring, and optimization of these models. In the following subsection, the models will be defined as a directed acyclic graph of the variables that uses the Shapley value as weight. By incorporating the Shapley value-based method and network analysis into the MLOps life cycle, organizations can enhance their understanding of model behavior, promote transparency, and optimize the performance of hierarchical machine learning models.

#### Network analysis for identifying key drivers of the model

Network analysis is a technique that can be used in MLOps to analyze the relationships between different variables or features in a machine learning model. The application of specific network analysis tools may help select key variables and intervention points in the graph that affect the model the most. If the contribution of a variable is added as weight to the network, it may alter the result of the analysis. By building a network, one can a) employ network science tools to understand the relationship of variables, c) determine the key nodes of the model, and b) select intervention points that significantly influence the output of the model.

The Shapley-weighted degree centrality may support the identification of the central position of a variable for their contribution to change *i.e*. has various connections throughout the model, and can be manipulated by several inputs, or can change the value of several outputs [67]. It is a simple measure of the number of neighbors of a node; however, it provides information on the degree one node influences or being influenced by other nodes. Degree centrality is defined as the number of edges incident on a node, and also considering the weight and directions:

$$\begin{array}{c} C_{d}\left(k\right)=\frac{\sum_{j=1}^{n}\left(w_{j,k}+w_{k,j}\right)}{2(n-1)},k\neq j \end{array}$$ (9)

where *C*_*d*_ defines the degree centrality measure for directed graphs, and *w*_*j*,*k*_ defines the weight of the edges (*e.g*. individual Shapley values) for the directed edge between node *j* and *k*, respectively.

Closeness centrality focuses on the average shortest path connections to other (significant) nodes, so with consideration, nodes with high closeness may be able to manipulate various key nodes at once [68]. It is defined as the reciprocal of the sum of the shortest path distances between the node and all other nodes in the network. For a node *x*_*k*_, the formula is as follows:

$$\begin{array}{c} C_{c}\left(k\right)=\frac{1}{\sum_{j\neq k}d\left(x_{j},x_{k}\right)} \end{array}$$ (10)

where *C*_*c*_(*k*) defines the closeness centrality, *x*_*k*_ is the node in question, *x*_*j*_ is a node in the network, *d*(*x*_*j*_, *x*_*k*_) is the shortest path distance between node *x*_*k*_ and node *x*_*j*_.

Betweenness centrality may improve the interpretation of the structure of the variables, as it measures the amount of shortest paths going through the node [69]. The shortest path may be influenced by the weights, so the role of this centrality is to find the measure of how dominant the role of the node is.

$$\begin{array}{c} C_{b}\left(k\right)=\sum_{x_{j}\neq x_{i}\neq x_{k}}\frac{\delta_{x_{j},x_{i}}\left(k\right)}{\delta_{x_{j},x_{i}}} \end{array}$$ (11)

where *C*_*b*_(*k*) defines the betweenness centrality, *x*_*j*_ and *x*_*i*_ denote nodes in the network, $\delta_{x_{j},x_{i}}\left(k\right)$ stands for the number of shortest paths from node *x*_*j*_ to node *x*_*i*_ that pass through node *x*_*k*_, and $\delta_{x_{j},x_{i}}$ is the total number of shortest paths from node *x*_*j*_ to node *x*_*i*_.

Other network analysis tools such as community detection (*e.g*. Louvian [70]) and shortest paths algorithms (*e.g*. Dijkstra’s algorithm [71]) may provide additional information on the relationship of the variables and significance of intervention points.

Network tools and the incorporation of the Shapley value allow us to identify the most influential variables, supporting the work of data scientists and modelers to focus on optimizing and fine-tuning the key drivers for improved model performance. With respect to the MLOps framework, network analysis offers a more holistic and systematic approach to understanding the underlying dynamics of complex machine learning models.

## Results

### Application of network and data analysis for model development

This section presents the processes for improving a system of environmental simulation based on structural equation models through a Hungarian case study provided by the Global Green Growth Institute [19]. The Green Growth Model of Hungary consists of three connected structural equation submodels, namely: energy, agriculture, forestry and other land use (AFOLU), water and waste models. To demonstrate of the efficiency of the data-driven Shapley value and network analysis for hierarchical models, we apply the methods on a submodel of the Hungarian water model. The introduced GGGI models rely on literature-based structural information and the model connections are validated with subject-matter experts. The model itself is deployed and maintained as a Python and Dash-based application. The practical and accurate development, monitoring, and maintenance of the ecosystem of the environmental models requires the principles of MLOps to apply.

As Hungary has progressed the least in SDG6 [5] since 2015, the interpretation of the water-related submodel (SDG6 indicators) could be of great help in explaining the required planning aspects of effective political interventions. Due to the fact that the modeling of the SDG indicators is a complex problem, the time series data (2000–2019) were compiled from sources such as FAO [72–76], NASA [77, 78], World Bank [79], United Nations [80], WHO & UNICEF [81], and the Hungary Ministry of Innovation and Technology [82] by GGGI. Some variables were imputed with the 2017 values due to unknown or missing data. The abbreviations and short description of the variables handled in the water model are shown in Table 2.

**Table 2:** Variables for the water efficiency model. The inputs and outputs contain data from 2000–2019. It is important to note that various inputs are imputed. (PI) denotes policy intervention points. Parameter (I) represents identifiable parameters. Variables without a specific unit are symbolized with a one in the Unit column of the table.

Notation	Abbreviation	Name	Unit	Type
${\hat{x}}_{1}$	AGVA	Agricultural Gross Value Added	$	Input
${\hat{x}}_{2}$	AIR	Agriculture area actually irrigated	1000 ha	Input
${\hat{x}}_{3}$	Arice	Area of Rice Paddy Irrigation	1000 ha	Input (PI)
${\hat{x}}_{4}$	CL	Cropland	1000 ha	Input
${\hat{x}}_{5}$	DW	Desalination Water	*m*^3^/year	Input
${\hat{x}}_{6}$	ERWR	External Renewable Water Resources	*m*^3^/year	Input
${\hat{x}}_{7}$	ETa	Actual Evapotranspiration	mm/year	Input
${\hat{x}}_{8}$	ETo	Evapotranspiration	mm/year	Input
${\hat{x}}_{9}$	GDPC	GDP per capita	$	Input
${\hat{x}}_{10}$	IGVA	Industrial Gross Value Added	$	Input
${\hat{x}}_{11}$	IRRTECHi	Irrigation technology proportion	1	Input (PI)
${\hat{x}}_{12}$	IRWR	Internal Renewable Water Resources	*m*^3^/year	Input
${\hat{x}}_{13}$	IWU	Industrial Water Withdrawal	10^9^*m*^3^/year	Input
${\hat{x}}_{14}$	Pop	Population	capita	Input
${\hat{x}}_{15}$	SGVA	Service Sector Gross Value Added Resources	$	Input
${\hat{x}}_{16}$	TW	Treated Wastewater	$/15*m*^3^	Input
${\hat{x}}_{17}$	WP	Water Price	$/15*m*^3^	Input
**x**_18_	AIRi	Irrigated area per irrigation technology type	1000 ha	Variable
*x*_19_	AWU	Agricultural Water Withdrawal	10^9^*m*^3^/year	Variable
**x**_20_	CI	Cropping Intensity	1	Variable
*x*_21_	Cr	Corrective coefficient	1	Variable
*x*_22_	ETc	Potential Crop Evaporation Vector	mm/year	Variable
*x*_23_	ICU	Irrigation Consumptive Use	mm/year	Variable
*x*_24_	IWR	Irrigation Water Requirement	10^9^*m*^3^/year	Variable
**x**_25_	IWRi	Irrigation Water Requirement per irrigation	1e9 *m*^3^/year	Variable
*x*_26_	IWW	Irrigation Water Withdrawal	10^9^*m*^3^/year	Variable
*x*_27_	MWU	Municipal Water Withdrawal	10^9^*m*^3^/year	Variable
*x*_28_	PAIR	Proportion of Irrigated Cropland	1	Variable
*x*_29_	TFA	Total Freshwater Available	*m*^3^/year	Variable
*x*_30_	TNCW	Total Non Conventional Water	*m*^3^/year	Variable
*x*_31_	TRF	Total Renewable Freshwater	*m*^3^/year	Variable
*x*_32_	TWW	Total Water Withdrawal	10^9^*m*^3^/year	Variable
*y*_1_	SDG 6.4.1	Water Use Efficiency	$/(*m*^3^/year)	Output
*y*_2_	SDG 6.4.2	Share of Freshwater Withdrawal to Freshwater Availability	%	Output
*θ*_20_	ICA	Cropland area actually irrigated (per crop type)	1000 ha	Parameter
*θ*_22_	Kc	Crop Factor (per crop type)	1	Parameter
*θ*_26_, *θ*_28_	IRRTECHEFFi	Irrigation efficiency per irrigation technology	%	Parameter
$\theta_{27_{1,2,3,4}}$	*β*_*MWU*_	Municipal Water Withdrawal coefficient vector	1	Parameter (I)
$\theta_{y_{2}}$	EFR	Environmental Flow Requirement	*m*^3^/year	Parameter

The structural equations of the water model is indicated in Table 3. The function indices are named after the variable indices. It is also indicated which final output the derived variable contributes to, as well as the equation of each function is specified as well as the literature-based evidence of the model relationships are added for each model equation.

**Table 3:** Structural model equations. The function indices are named after the variable indices. $\hat{\theta }$ defines parameters that ensure the matching of dimensions or any physical conversions or attributes. The source of model equation is also indicated in the table.

Function	Derived variable name	Relevant to	Equation	Reference
*f*_18_	Irrigated area per irrigation technology type (AIRi)	SDG 6.4.1, SDG 6.4.2	$x_{18,i}={\hat{x}}_{11,i}{\hat{x}}_{2}$	[83]
*f*_19_	Agricultural Water Withdrawal (AWU)	SDG 6.4.1, SDG 6.4.2	*x*_19_ = *x*_26_	[84]
*f*_20_	Cropping intensity (CI)	SDG 6.4.1, SDG 6.4.2	$x_{20,i}=\frac{\theta_{20,i}}{{\hat{x}}_{2}};i=1,\ldots ,\left|\theta_{20}\right|$	[85]
*f*_21_	Corrective coefficient (Cr)	SDG 6.4.1	*x*_21_ = 1/(1 + (*x*_28_/(1 − *x*_28_) * *θ*_21_))	[86]
*f*_22_	Potential Crop Evaporation Vector (ETc)	SDG 6.4.1, SDG 6.4.2	$x_{22}=\theta_{22}\cdot x_{20}\cdot {\hat{x}}_{8}$	[85]
*f*_23_	Irrigation Consumptive Use (ICU)	SDG 6.4.1, SDG 6.4.2	$x_{23}=\left|x_{22}-{\hat{x}}_{7}\right|$	[85]
*f*_25_	Irrigation Water Requirement per irrigation (IWRi)	SDG 6.4.1, SDG 6.4.2	$x_{25,i}=\hat{\theta }x_{23}x_{18,i}$	[85]
*f*_26_	Irrigation Water Withdrawal (IWW)	SDG 6.4.1, SDG 6.4.2	$x_{26}=\sum_{i=1}^{|x_{25}|}\left(x_{25,i}/\theta_{26,i}\right)+{\hat{x}}_{3}{\hat{\theta }}_{\text{rice\_height}}$	[84]
*f*_27_	Municipal Water Withdrawal (MWU)	SDG 6.4.1, SDG 6.4.2	$x_{27}=e^{\theta_{27_{1}}}{\hat{x}}_{17}^{\theta_{27_{2}}}{\hat{x}}_{9}^{\theta_{27_{3}}}{\hat{x}}_{14}^{\theta_{27_{4}}}10^{-9}$	[87]
*f*_28_	Proportion of Irrigated Cropland (PAIR)	SDG 6.4.1	$x_{28}=\left(\sum_{i=1}^{|\theta_{28}|}\theta_{28,i}\right)/{\hat{x}}_{4}$	[86]
*f*_29_	Total Freshwater Available (TFA)	SDG 6.4.2	*x*_29_ = *x*_30_ + *x*_31_	[88]
*f*_30_	Total Non-Conventional Water (TNCW)	SDG 6.4.2	$x_{30}={\hat{x}}_{5}+{\hat{x}}_{16}$	[88]
*f*_31_	Total Renewable Freshwater (TRF)	SDG 6.4.2	$x_{31}={\hat{x}}_{6}+{\hat{x}}_{12}$	[88]
*f*_32_	Total Water Withdrawal (TWW)	SDG 6.4.1, SDG 6.4.2	$x_{32}=x_{27}+x_{19}+{\hat{x}}_{13}$	[88]
$f_{y_{1}}$	Water Use Efficiency (SDG 6.4.1)	SDG 6.4.1	$y_{1}=\left({\hat{x}}_{1}\left(1-x_{21}\right)+{\hat{x}}_{10}+{\hat{x}}_{15}\right)/\left(x_{32}10^{9}\right)$	[86]
$f_{y_{2}}$	Share of Freshwater Withdrawal to Freshwater Availability (SDG 6.4.2)	SDG 6.4.2	$y_{2}=x_{32}/\left(x_{29}-\theta_{y_{2}}\right)10^{2}$	[88]

**Fig 3:**
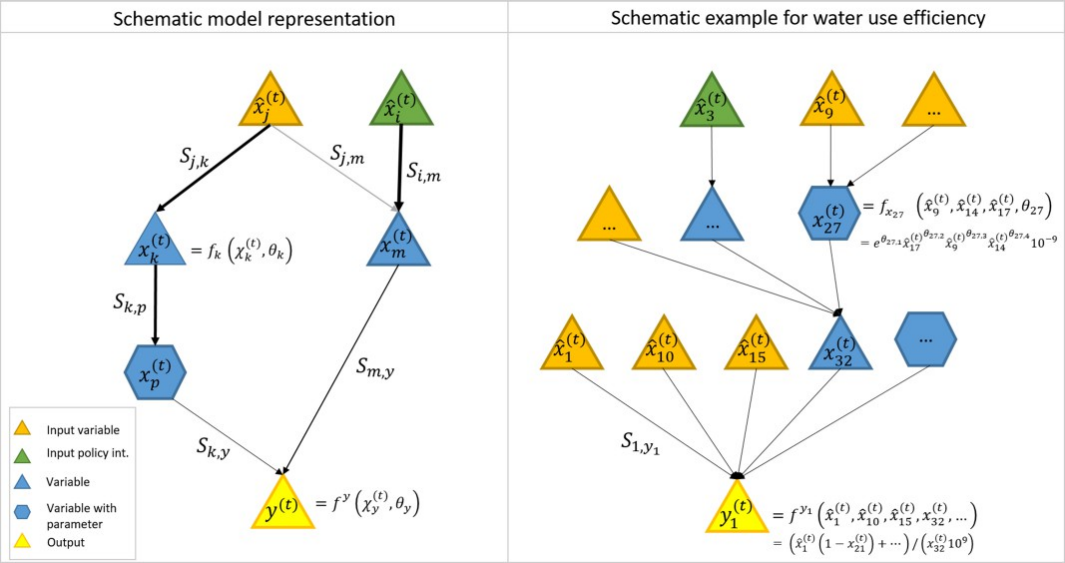
Schematic representation of the Shapley value-based network interpretation of structural equation model and variable contributions.

In Fig 3, the schematic representation of the Shapley value-based interpretation of the structural equation model and the contributions of the variables is shown. The structured equation and the Shapley value method are formulated as follows.

In the following sections, we focus on the validation of the hierarchical models to show that such models provide correct information and knowledge to transfer to decision-makers. As the MLOps principles focus not only on the development but also on validation and maintenance, they contain the necessary toolset to confirm the possible use of the models. Therefore, first, we validate the model by comparing it with linear regression and k-th nearest neighbors models to ensure the validity of the connection between the independent and the dependent variables. Then the roles of the variables are discussed with the help of Shapley-value-based analysis. Lastly, we introduce network analysis to select the most important input.

#### MLOps life cycle in environmental model development

The three stages of the MLOps life cycle include the business and data understanding, model development, and model operation phases. Continuous revision, development, and evaluation of environmental models are required to ensure adaptation to spatial and temporal changes. Reviewing and analyzing the model can help ensure that it is aligned with the problem statement and the data requirements. This includes reviewing the policy needs and requirements, identifying country specifications, data sources, and quality, as well as changes over time to ensure that the model is designed to meet the desired SDG outcomes. Therefore, the modeling development phase includes optimization of the model parameters, validation of the model against new data, and integration of new knowledge and insights into the model.

The GGSim model aims to predict several sustainable development goals. The water submodels include the water use efficiency (SDG 6.4.1) and share of freshwater withdrawal to freshwater availability (SDG 6.4.2) as outputs. As the Shapley value evaluates the contribution of variables to the model output, first, the accuracy of the models should be evaluated. The predictions are evaluated for the GGSim model, two machine learning techniques, namely linear regression and k-th nearest neighbors, and the parameter optimization.

**Fig 4:**
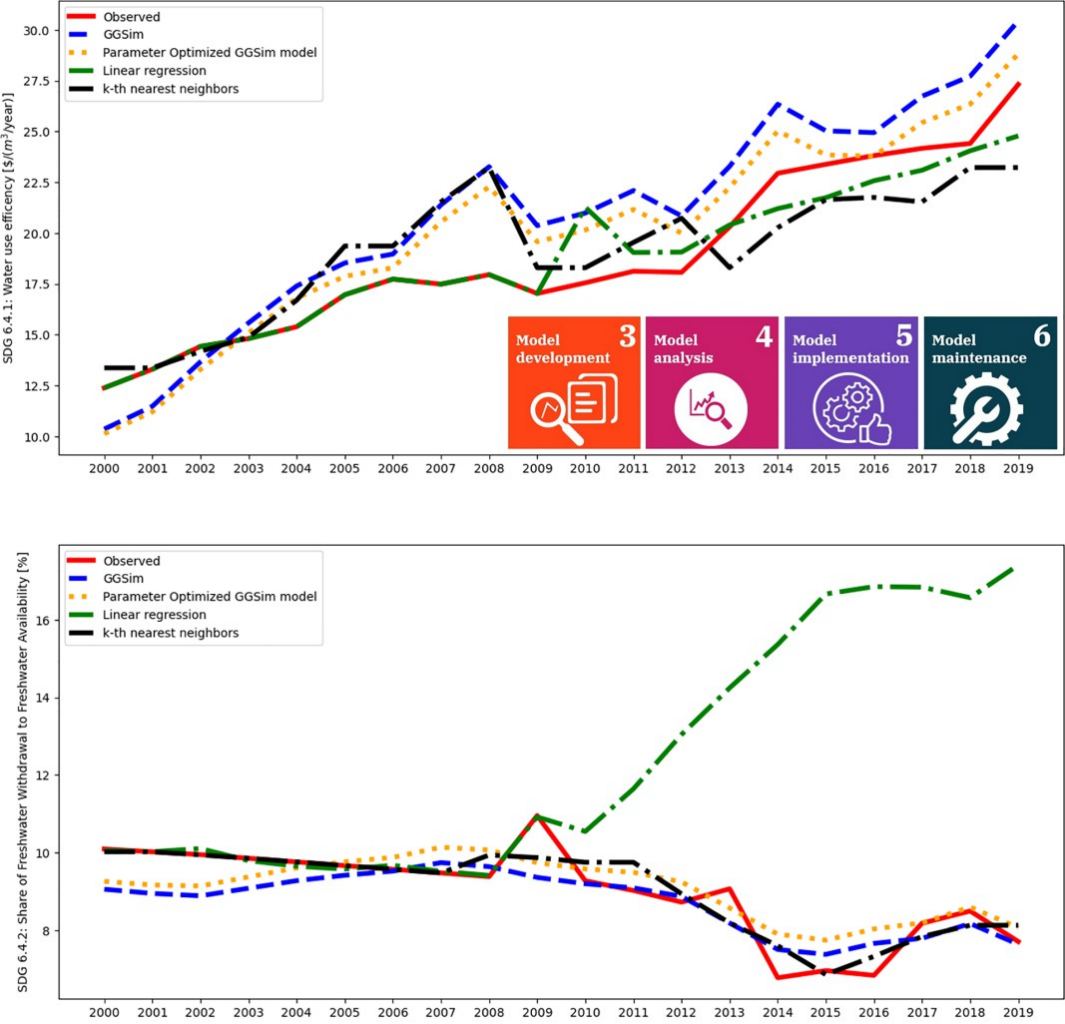
Prediction of the GGSim model against observed data. The expert-built model (SDG 6.4.1 *r*^2^: 0.519, SDG 6.4.2 *r*^2^: 0.639) is also compared against a linear regression (SDG 6.4.1 *r*^2^: 0.907, SDG 6.4.2 *r*^2^: -20.46) and a k-th nearest neighbor algorithm (SDG 6.4.1 *r*^2^: 0.91, SDG 6.4.2 *r*^2^: 0.69). The k-th nearest neighbor shows the most promise, however, one cannot easily explain the inner structure of the black-box model. The parameter optimization of the municipal water withdrawal equation improved the model (SDG 6.4.1 *r*^2^: 0.744, SDG 6.4.2 *r*^2^: 0.65).

Fig 4 presents the results of different models for the efficiency of water use (SDG 6.4.1) and the share of freshwater withdrawal to freshwater availability (SDG 6.4.2) SDG indicators. The red line represents the observed data, whereas the blue dashed line shows the output of the GGSim model, which was built by experts. It seems that the error is somewhat systematic; however, some variables have no variance due to data imputation. Thus, the systematic error may be associated with a lack of adequate data. The *r*^2^ was also calculated: for SDG 6.4.1 *r*^2^: 0.519, SDG 6.4.2 *r*^2^: 0.639. Additionally, parameter optimization was performed on the GGSim model for municipal water use. The SDG 6.4.1 model improved significantly, with a *r*^2^ of 0.744, while SDG 6.4.2 only had an insignificant change in fitness (*r*^2^ = 0.65).

Two machine learning models have been applied to the input data to predict the output while leaving out intermediate (derived) variables. Linear regression and the k-th nearest neighbor [89] models were trained every second year, starting from 2000 to 2009. It seems that linear regression cannot capture the complexity of the computation for SDG 6.4.2, and its *r*^2^ is -20.46, while the SDG 6.4.1 model provides an acceptable fit (0.907). Although the coefficients are known, the white-box nature cannot be used due to the failing prediction of SDG 6.4.2. However, the k-th nearest neighbors method was able to fit a well-performing model on the observed output variable (SDG 6.4.1 *r*^2^: 0.91, SDG 6.4.2 *r*^2^: 0.69). Due to the black-box nature of the model, one may not be able to interpret why it provides predictions as such, but the improvement in accuracy reassured the connection between the inputs and outputs. If the model is hierarchical, it may also be hard to interpret; in some cases, the conversions and calculations are not trivial; therefore, it requires a method that is capable of describing the contributions between the variables.

**Fig 5:**
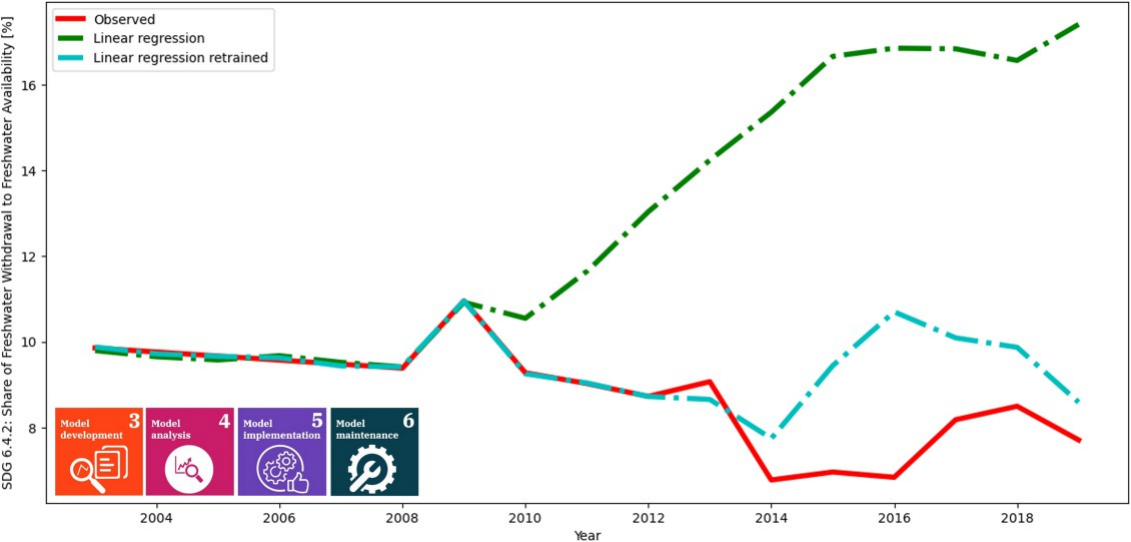
Retrained linear regression for SDG 6.4.2. The red line depicts the observed variable, the green one represents the linear regression from Fig 4, and the cyan colored one represent the retrained linear regression. The years before 2003 were dropped, and the model was retrained on years 2003–2012. The “new” model seems to fit better on the observed model, proving the necessity of continuous maintenance for machine learning models.

This example has revealed the necessity for continuous model development and maintenance through data-driven machine learning techniques to ensure the quality of model performance. As is evident from the application of linear regression and k-th nearest-neighbor machine learning models in a given scenario, the model accuracy can vary significantly. Models should be trained over time to adapt to changes. It includes updating model parameters and incorporating new data, and variables, thereby improving the predictive capability of the model. The application of the MLOps-based continuous development approach is a must to ensure that the models capture the complexity of the sustainability problems and iteratively enhance accuracy and robustness and provide more reliable predictions. Therefore, we re-trained linear regression for SDG 6.4.2, which is presented in Fig 5. Here, the red line represents the observed variable, the green line represents the previous linear regression, and the cyan line represents the retrained linear regression. During retraining, we dropped the years from 2000–2002, and trained on from 2003 to 2012. The regression predicts the whole time interval. The new prediction seems to be closer to the observed data than the previous linear regression.

Emphasizing the significance of validated model structures is essential for performing in-depth analysis and model development. By focusing on validated models, data scientists and modelers can confidently investigate the underlying dynamics without concerns about flawed or unreliable outcomes. The reliable model structure facilitates the analysis of variable contributions to the model output and the identification of potential policy intervention points.

#### Direct and indirect contribution of the variables to the model output

Understanding the direct contribution of variables provides insight into which variables have the most significant impact on the output of the model. This information is crucial to identify the key drivers of the system being modeled, which can be used to inform decision-making and policy development. The indirect contribution of a variable refers to the impact that a variable has on the output of the model through its interactions with other variables in the network, which can allow us to identify complex relationships. During model validation, it is imperative to ensure that only the relevant variables remain part of the model. The Shapley value heavily relies on the role of the variable in the model and the variance of the data, and so ensuring variance may define the importance of a variable.

**Fig 6:**
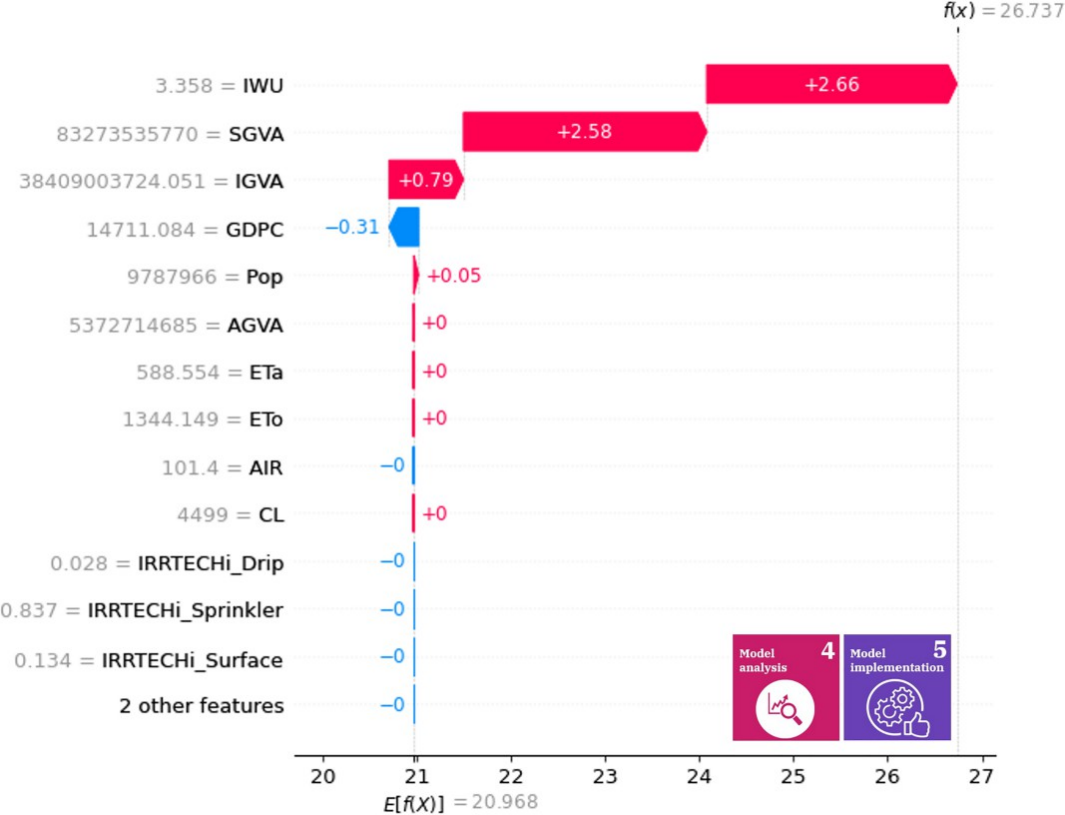
Indirect contribution of variables for SDG 6.4.1 in year 2017. The dataset contains data imputations that were made with the 2017 data, therefore, this year is the best candidate for Shapley analysis. The X-axis shows the function value of SDG 6.4.1 in 2017, while the y axis presents how one variable changes the expected value of the function.

**Fig 7:**
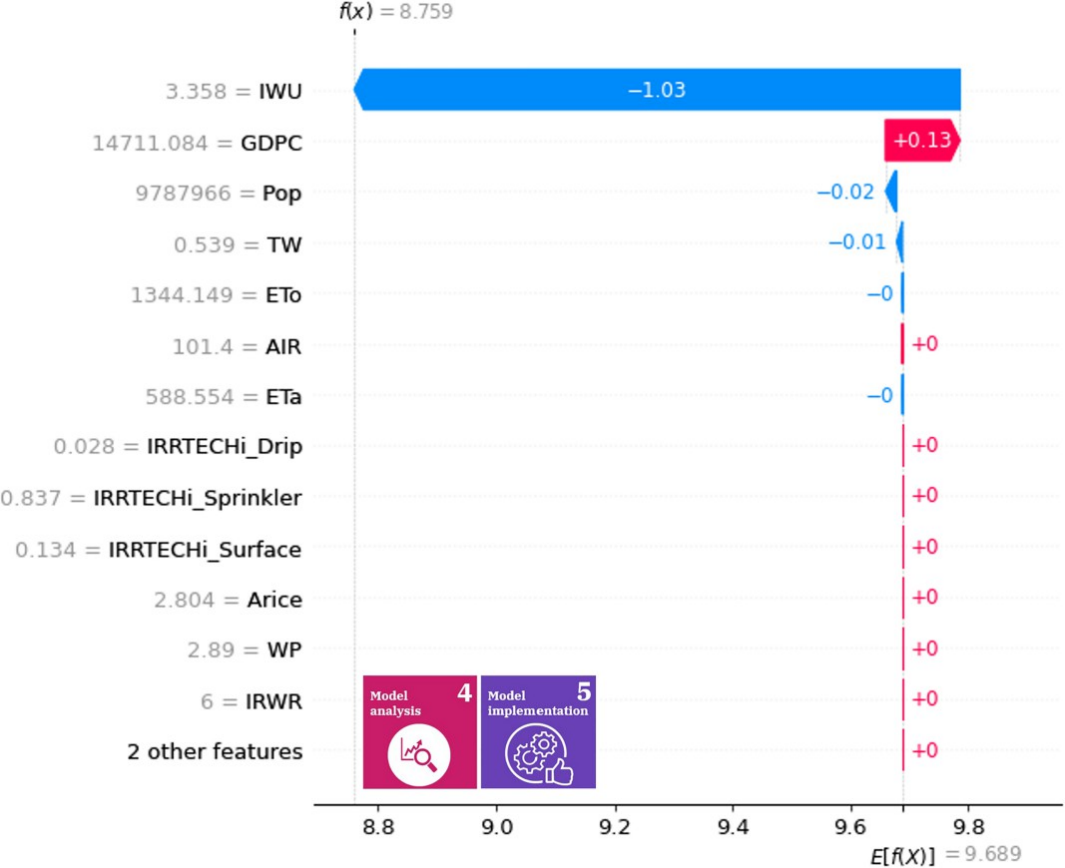
Indirect contribution of variables for SDG 6.4.2 in year 2017. The X-axis shows the function value of SDG 6.4.2 in 2017, while the y axis presents how one variable changes the expected value of the function.

Figs 6 and 7 illustrate the indirect contribution of variables to water use efficiency and water stress level in 2017. It is advantageous to visualize how one variable changes the expected value; the impact of one particular year may help in deciding what policies should be implemented next year. The cumulative effects on the predictions can be measured, which provides information on how different variables may interact with each other. In this representation, the negative contribution values should not be associated with negative correlations to the output. The water use efficiency model is biased towards industrial water withdrawal (IWU), and service sectoral gross value added resources (SGVA), the data for 2017 indicate that most variables have no proper impact on the output of the model, except for what is required for the calculation of the expected value and conversions. For water efficiency (SDG 6.4.1), industrial water withdrawal, service sector gross value added resources, industrial gross value added (IGVA) and GDP per capita (GDPC) can be considered high-impact variables (Fig 6). For the level of water stress (EW2), industrial water withdrawal and GDP per capita are the drivers of contribution from the point of view of the SDG output indicator (Fig 7).

In the case of the water efficiency and water stress submodels, the decisive input in 2017 was the withdrawal of industrial water in Hungary; therefore, in order to improve the achievement of the 2030 Agenda, it is necessary to identify political interventions that reduce the value of this input, since the effect of IWU strongly affects the performance of SDG 6.4.1 & 6.4.2 indicators. However, due to the complexity of the SDG indicators, disaggregated inputs [90] are easier to handle. The reduction of water footprint [91], the circularity of water [92], and the nature-based solutions [93] can be effective tools to support SDG6.

By applying the framework we propose, it is possible to analyze the entire model structure, which also laid the foundation for the validation of the elements of the model. It is important to emphasize that all models included in this manuscript have undergone expert validation.

It is important to note that the effectiveness of individual state variables (policy intervention points) can vary depending on the current value of the other inputs, so policy monitoring is also an important task, which can be supported by our proposed Shapley value-based management. If it is not the given annual contributions but the tendentious driving forces that need to be identified in the SDG framework, the indirect mean Shapley values can be called upon.

**Fig 8:**
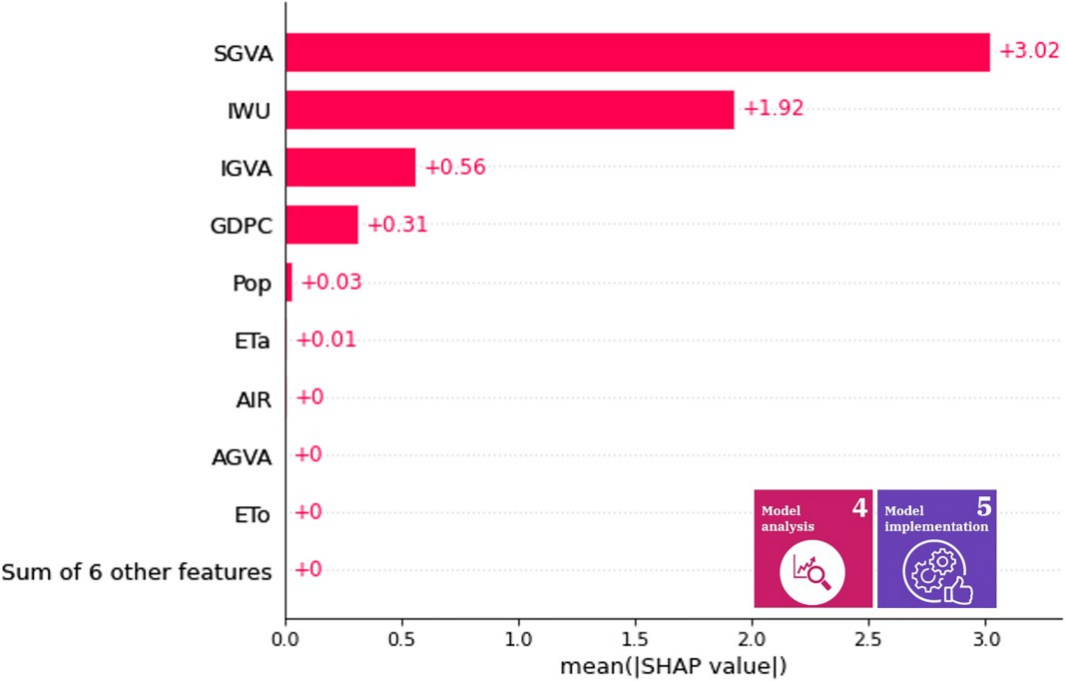
Indirect mean Shapley values of variables for SDG 6.4.1. The X-axis shows the average of caused (relative) change in the SDG 6.4.1 from 2000 to 2019, while the y axis presents the average impact of a variable.

**Fig 9:**
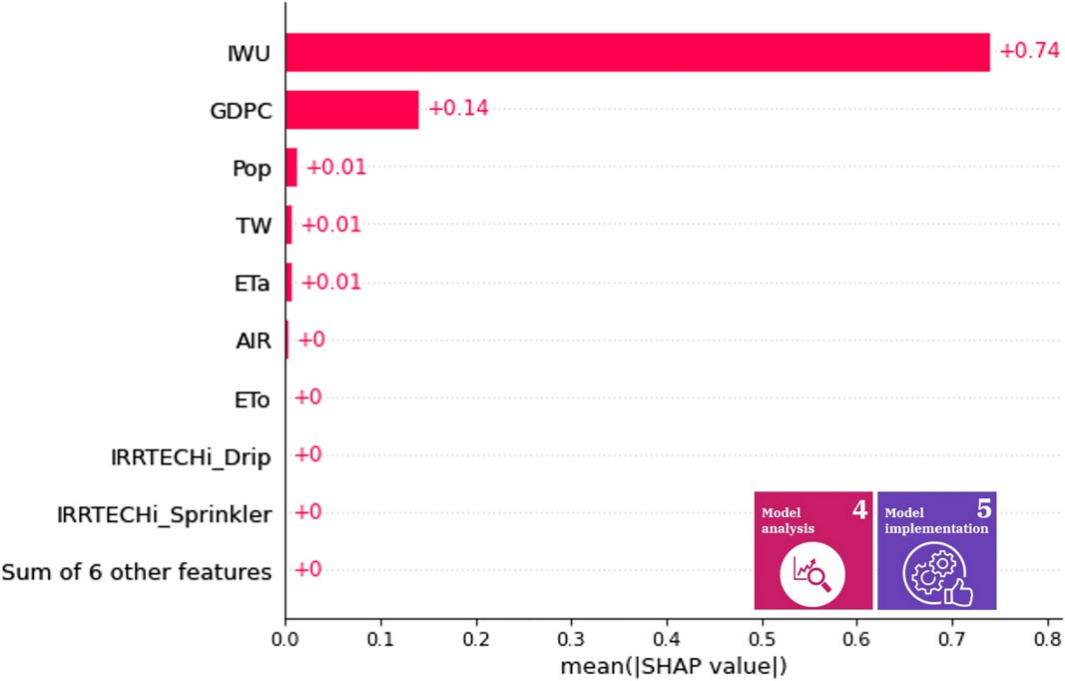
Indirect mean Shapley values of variables for SDG 6.4.2 the level of water stress. The X-axis shows the average of caused (relative) change in the SDG 6.4.2 from 2000 to 2019, while the y axis presents the average impact of a variable.

The indirect mean Shapley values of the variables for the efficiency of water use are illustrated in Fig 8 and for the level of water stress in Fig 9. The mean contribution determines the significance of a variable over the years, so these mean contributions can be used as a general rule of thumb during the selection of appropriate policies. The high impact variables are similar compared to the 2017 data. Therefore, we can deduce that the roles do not differ significantly over time. In decision support, this representation may help in selecting features with high impact over time, model interpretation (by defining the high-impact variables), and validation. For example, if one variable relevance is a scientific fact and the mean absolute Shapley value is insignificant, then the model may have flaws that must be corrected before the actual information visualization is provided to the decision makers.

Fig 8 shows that the average contribution of the service sector to water efficiency is the most significant. This indicator indirectly characterizes tourist activity, the operation of hotels, restaurants, laundries, *etc*., thus affecting a wide range of water uses, and in SDG12 for the co-benefits that appear through food waste [94]. This high Shapley value draws attention to the fact that the potential of the service sector for sustainable watershed management in Hungary is worth considering as an effective point of political intervention.

The indirect mean Shapley values of the variables for the level of water stress are illustrated in Fig 9. The decisive role of industrial water withdrawals in water stress is outstanding when examined over the entire period. In this case, the importance of productivity developments comes to the fore, which already show a decreasing trend in water stress in several developed countries [95]. Water stress is an excellent example of the fact that SDG indicators are difficult to directly regulate, so the identification of intervention points for which effective political measures [96] can be formulated is essential for the implementation of the 2030 Agenda.

**Fig 10:**
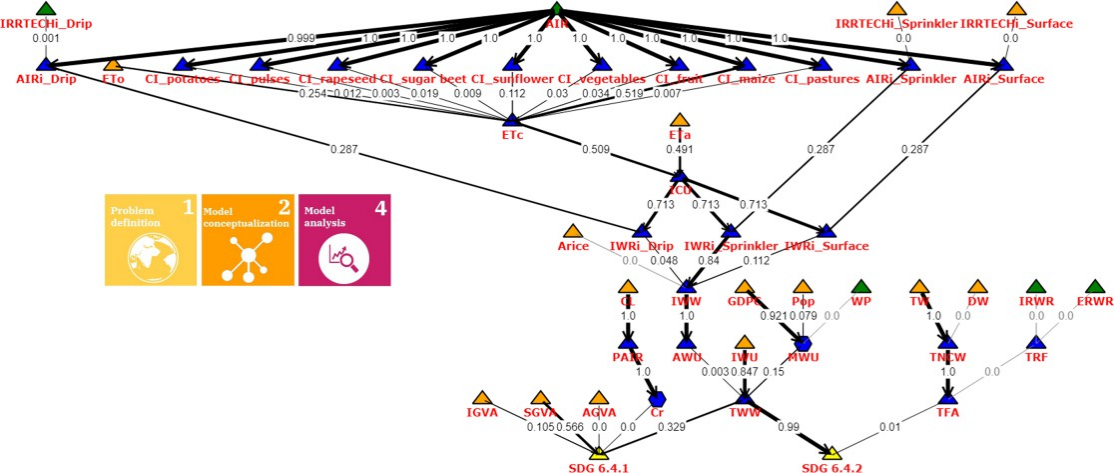
Direct mean contribution of variables to the change in the output variables. The types of nodes are represented with different colors and shapes (green triangle—policy intervention points; orange triangle—input variables; blue triangle—variables; blue hexagon—variables with parameters; yellow triangle—output). The thickness of the arrows represents the strength of direct variable contribution.

**Fig 11:**
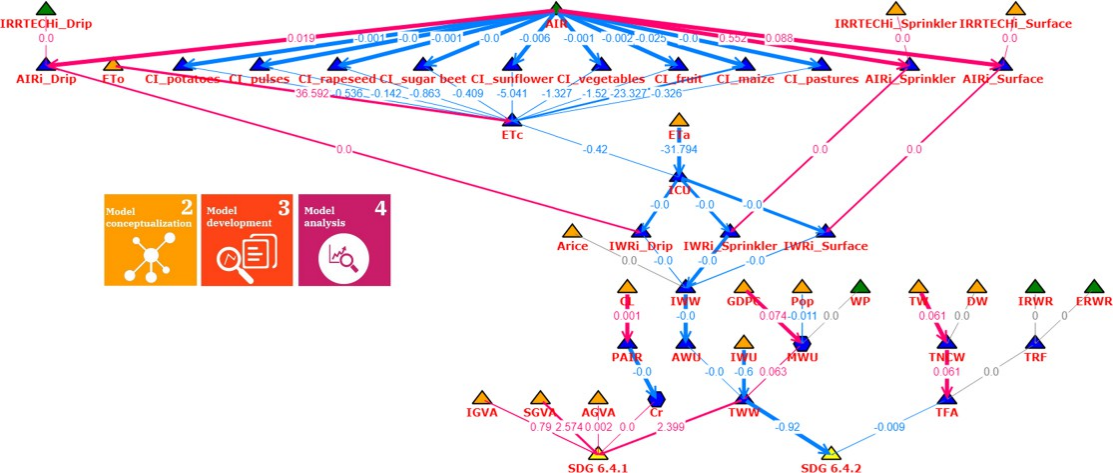
Direct contribution of variables to the change in the output variables for 2017. The type of nodes are represented with different colors and shapes (green triangle—policy intervention points; orange triangle—input variables; blue triangle—variables; blue hexagon—variables with parameters; yellow triangle—output). The thickness of the arrows represents the strength of direct variable contribution.

The direct contribution of the variables to the changes in the output variables is shown in Figs 10 and 11. Nodes are differentiated by their color and shape, with policy intervention points represented by green triangles (${\hat{x}}_{3}$, ${\hat{x}}_{11}$), input variables by orange triangles (${\hat{x}}_{k},k=1,\ldots ,17$), derived variables by blue triangles (*x*_*k*_, *k* = 18, …, 32), variables with (possibly identifiable) parameters by blue hexagons (*x*_21_, *x*_27_), and output SDG indicators by yellow triangles (*y*_1_, *y*_2_). The thickness (the greater the better) of the arrows represents the strength of direct variable contribution (the width is proportional to the individual to total contribution ratio). Here, the percentage of the total average contribution is provided for each arrow with respect to all inputs of a model. The gray arrows depict the zero contribution.

Figs 10 and 11 show that there are aggregated and raw data input sources to describe the aggregated SDG indicators, which can be used to identify optimal intervention points (policies) based on their systematic exploration and impact of the contribution on the SDG output, and this systematic exploration can also be used to check the effectiveness of existing policies.

Fig 10 shows that irrigated agricultural areas (AIR) influence irrigation water use (IWW) through crop specifications (ETc, ICU), which in relation to total water withdrawal (TWW) affects the level of water stress (SDG 6.4.2) the most. It seems that the water price (WP), the internal and external renewable water resources (IRWR, ERWR) as political intervention points did not have a significant impact in the period examined based on Hungarian data, so it is more expedient to develop the service sector instead (SGVA), or improve water retention (ETa), more precisely in developed and scheduled irrigation solutions, such as the internet of things and wireless sensor network technologies [97]. Based on the model structure, the other option is to reduce industrial water use, which can be supported by moving toward water reuse and recycling [98] and technological solutions saving water [99]. The role of the variables can also be analyzed in a yearly breakdown, which lays the foundation for a better understanding of the dynamics of the SDG indicators and their input data sources. The annual contribution of the variables in 2017 can be seen in Fig 11.

The values on the edges show the contribution relative to the value of the output variable of the model, so if the change in the output is low, the relative contribution values on the edges are also low. However, the thickness of the edges symbolizes the degree of contribution of the given variable, so the absolute and relative effects can be read together for all SDG indicators included in the model. As can be seen in Fig 11, the effect of the progress achieved in industrial water use on both SDG6 indicators is even more pronounced than in the case of the mean time series contribution (Fig 10).

Conducting the analysis of the direct and indirect contribution of the variables to the model output reveals key information regarding the drivers of the models, which serves as validation of the developed model, a base for further improvement, and enabling informed decision-making and selection of key policy intervention points through understanding the underlying interactions within the variables and the model output.

#### Selection and validation of policy intervention points

**Fig 12:**
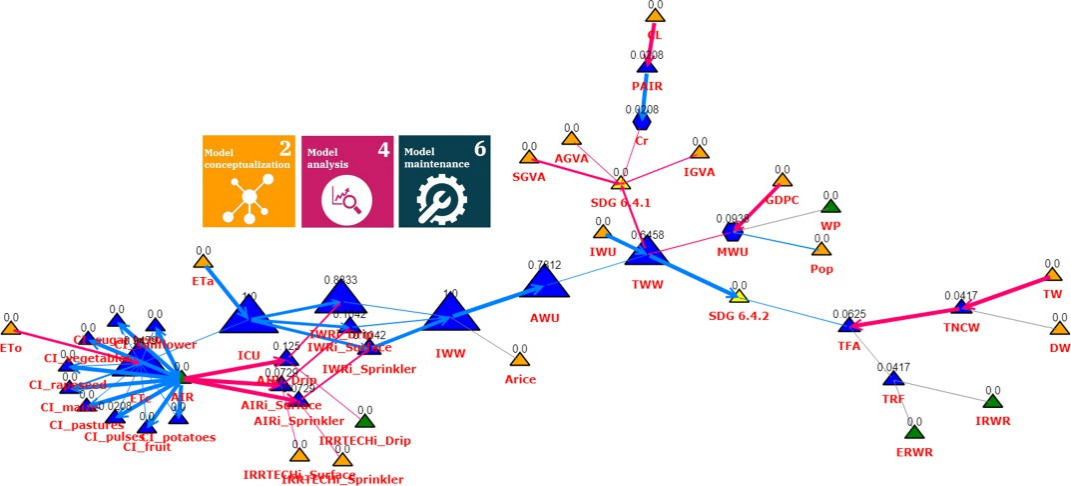
Network representation of the betweenness centralities for the SDG 6.4.1 & SDG 6.4.2 indicators in water model. The size of the nodes presents the importance of centralities. The types of nodes are represented with different colors and shapes (green triangle—policy intervention points; orange triangle—input variables; blue triangle—variables; blue hexagon—variables with parameters; yellow triangle—output). The thickness of the arrows represents the strength of the direct variable contribution.

The selection and validation of policy intervention points is an important step in the development of effective policies and interventions that can improve outcomes in the water model. We utilized the potential of sensitivity and network analysis to validate policy intervention points and to support the selection of possible ones. By testing the sensitivity of the model to changes in the value of intervention points, we can identify the points that have the greatest potential to improve outcomes. This information can be used to prioritize interventions and focus resources on the most effective intervention points. The tools of network science enable us to identify critical paths and nodes within the water model, based on which information we can focus on the most important variables in the system, and develop interventions that target these variables. Fig 12 shows the betweenness centrality of network water efficiency (6.4.1) and water stress (6.4.2) SDG indicators.

In the network of Fig 12, the size of the nodes is the same as the degree of their intermediary role (betweenness centrality measure), so the larger nodes represent successful mediators, i.e. potential political interveners, while the influence of the smaller nodes is less significant in the state changes of the SDG system of the water model. Regarding the intermediary role, in the case of the SDG6 indicators, crop evaporation (Etc), irrigation (ICU, IWW), agricultural water use (AWU) and total water withdrawal (TWW) play a prominent role in the network, so a direct or indirect reduction of these node values may mean political implementation potential. Based on the example shown in Fig 12, it can also be seen that if the used state variables e.g. water withdrawals cannot be reduced directly, the model extension with additional politically perturbable variables can be performed based on the network science-based model analysis.

Utilizing network science tools facilitates the identification and validation of crucial policy intervention points, a key aspect of model validation and development. By employing these tools, researchers and decision-makers can rigorously verify the effectiveness and reliability of proposed policy changes before implementing them in real-world scenarios, ensuring a more robust and informed decision-making process.

## Discussion

The proper translation of information empowers decision-makers. The decision support often relies on the visualization of data and models. Dashboards with incorporated visualizations and hierarchical structures can help in identifying trends, relationships between variables, sensitivities of features *etc*. Knowledge, insight, and information transfer is required to improve the decision-making capacity of stakeholders [100]. The role of explainable AI is to provide knowledge transfer, when the actual knowledge is hidden [101]. Shapley-based networks have already been researched [102], however, we focus on their implications for decision-makers and their connection with MLOps practices. We must note that the Shapley values may not be interpretable if the data is incorrect, the model acts on incorrect assumptions, has inappropriate biases, or the incorrect set of variables is used as inputs [103], which is why checking and validating the prediction ability of the models is crucial before understanding their structure. The accuracy of model evaluation relies heavily on the quality of the underlying data, and poor data quality can result in inaccurate assessments of model performance and variable effects. Additionally, the need for generally accepted data sources is emphasized, especially when dealing with diverse economies or environmental backgrounds. Furthermore, addressing structural flaws in models is a key focus of MLOps, which involves retraining existing models or creating new ones by incorporating new variables and insights.

However, the method that facilitates the identification of potential intervention points cannot be utilized to propose exact policies. Furthermore, the method was developed to support decision-makers rather than substitute them. Also, the proposed case study is an isolated submodel of the whole SDG framework, and the change of one variable may seem to be very effective; it may negatively influence other areas and models, provided that the models are connected. Consider the following counterfactual: removing rice paddies may decrease water use and improve water use efficiency; however, the harvest will also be reduced, and so it will affect the food market. In other words, water use is improved, but the number of starving people can increase due to costly imports and reduced harvests. Therefore, the selection of the policy intervention is a great balancing act. The change in one variable may initiate a trade-off between several of the outputs, even if the models may not account for it. Furthermore, for one country in a global economy, it may seem reasonable to take actions, *e.g*. reduce rice production, if all countries do the same, which may affect the global food supply. Therefore, there is a need to evaluate policy-making in a system of systems settings.

Regarding the evaluation of sustainability models with Shapley-based network analysis, the results present a handful of irrelevant variables to the change in prediction. Namely, water price, rice paddy irrigation area, and internal and external renewable water resources lack variance, therefore they have no impact. The agricultural gross value added (AGVA) also has a minuscule contribution due to its small size compared to its industrial (IGVA) and service sectoral (SGVA) versions. Generally speaking, agriculture draws the most water worldwide (about 69%) [104], due to crop and animal needs, and therefore plays a significant role in feeding the population. The output shows a one-sided picture of water use. The description of water use efficiency can be understood as the economic value per cubic meter of water [105]. Therefore, the interpretation may have methodological flaws [106], as it may not consider other important factors. The SDG indicator may be related to behavioral factors that impact municipal water withdrawal [104]. Here, the water price indicator can be used as an incentive to control behavior, yet this variable does not have variance in the Hungarian data set provided by the GGGI. However, the price of water was selected as an intervention point in the original model.

The application and key scientific contribution of the MLOps methodology presented in the research are two-fold since the experts who create the models can represent the entire model structure, therefore it is possible to validate the model features. In addition, our proposed methodology also supports problem exploration and understanding through data. In other words, data-based qualitative validation and network science, as well as Shapley value-based qualitative validation, can be implemented together, thereby effectively supporting modeling processes.

## Conclusion

This paper emphasizes the importance of developing life cycle-based models that incorporate network and data analysis practices in sustainability planning, as demonstrated through an application study of the Hungarian water model developed by the Global Green Growth Institution. To accurately track progress toward achieving the Sustainable Development Goals (SDGs), it is necessary to use data-driven models that are country-specific and aligned with policy interventions. However, modeling such complex relationships is often difficult due to the diverse scale and quality of data, policy initiatives, and economic and social developments across countries. Continuous life cycle-based revision, analysis, development, and evaluation of environmental models are needed to ensure model adaptation to spatial and temporal changes.

Therefore, we propose the utilization of tools based on network and data science throughout the modeling life cycle to ensure more accurate models to assess the SDGs and evidence-based policy-making. The potential of Shapley value has been introduced to identify key drivers of the system being modeled and to support decision-making and validate policy interventions, which facilitates understanding of the direct and indirect contribution of variables. This enables greater insight into which variables have the most significant impact on the output of the model (SDG indicators). Furthermore, we suggest using a Shapley value-driven sensitivity analysis of the changes in intervention point values, and network analysis to identify critical paths and nodes in the selecting and validating of policy intervention points. Ensuring the accuracy of model evaluation is imperative, hinging on the detailed handling of underlying data quality to prevent incorrect conclusions about the model’s performance. It is crucial to establish homogeneous, generally accepted samples, particularly when considering diverse economic and environmental backgrounds, underscoring the need for a clear definition in the modeled area. Future development plans should prioritize advancements in model evaluation by refining methodologies for handling data, while also focusing on the continuous innovation of MLOps to proactively identify and rectify structural flaws in models through either retraining existing models or creating new ones with updated variables and insights.

The real-life application study of network and data analysis for the development of the Hungarian water model underlines the efficiency of the proposed aspects. We believe that the proposed data-driven life cycle management of sustainability models has great potential in real-life sustainability planning and decision-making at any administrative level including model identification, development and validation.

## Supporting information

S1 Fig (TIF)

S2 Fig (TIF)

S3 Fig (TIF)

S4 Fig (TIF)

S5 Fig (TIF)

S6 Fig (TIF)
