# Supplementary figures and images for: Network science and explainable AI-based life cycle management of sustainability models
Source: PLoS One. 2024 Jun 13;19(6):e0300531. doi: 10.1371/journal.pone.0300531 (PMC11175538; doi:10.1371/journal.pone.0300531)

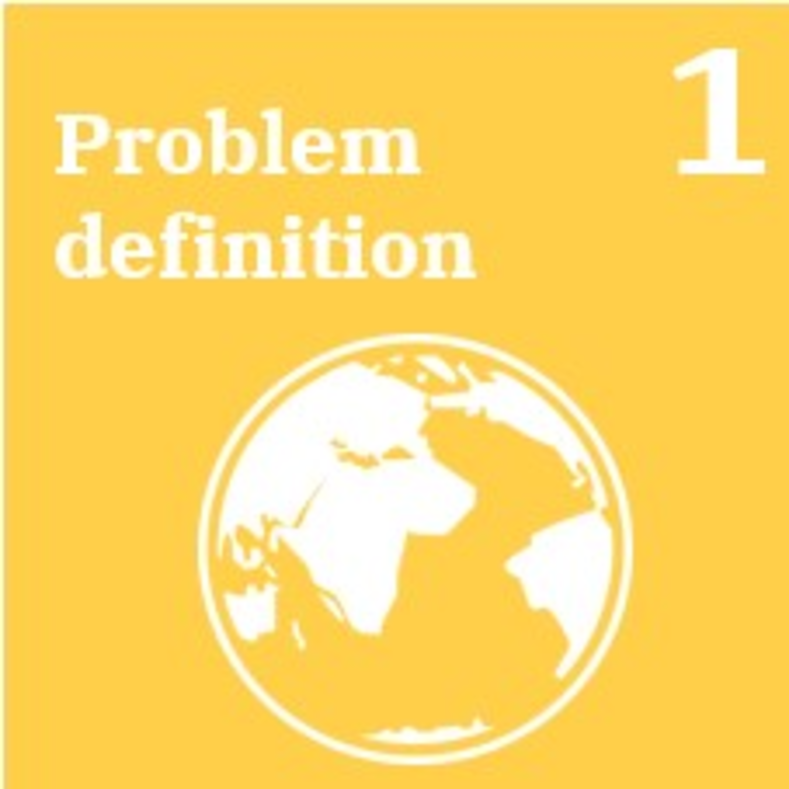

Supplement: S1 Fig — (TIF) [file pone.0300531.s001.tif]

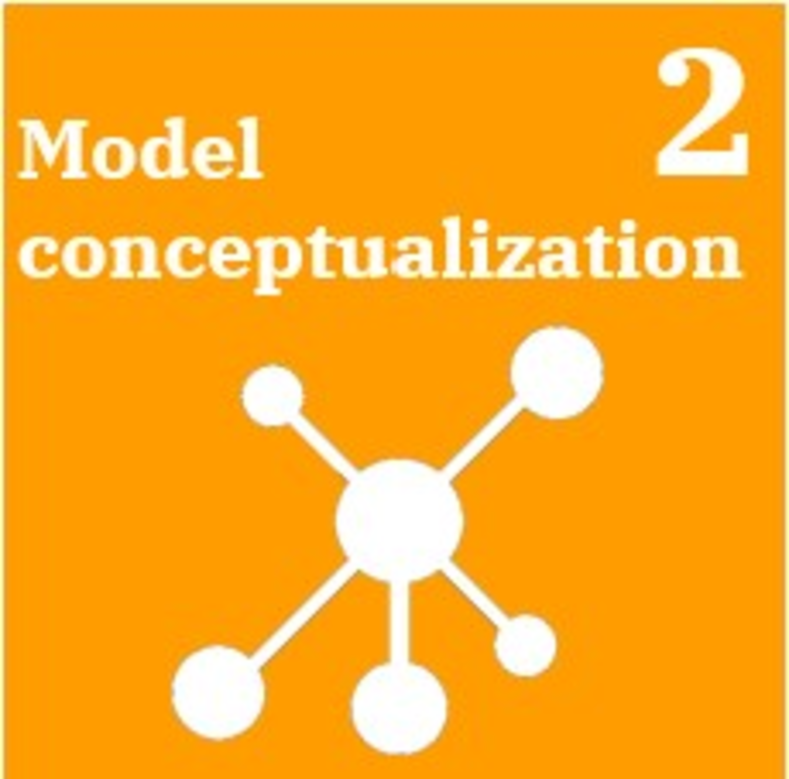

Supplement: S2 Fig — (TIF) [file pone.0300531.s002.tif]

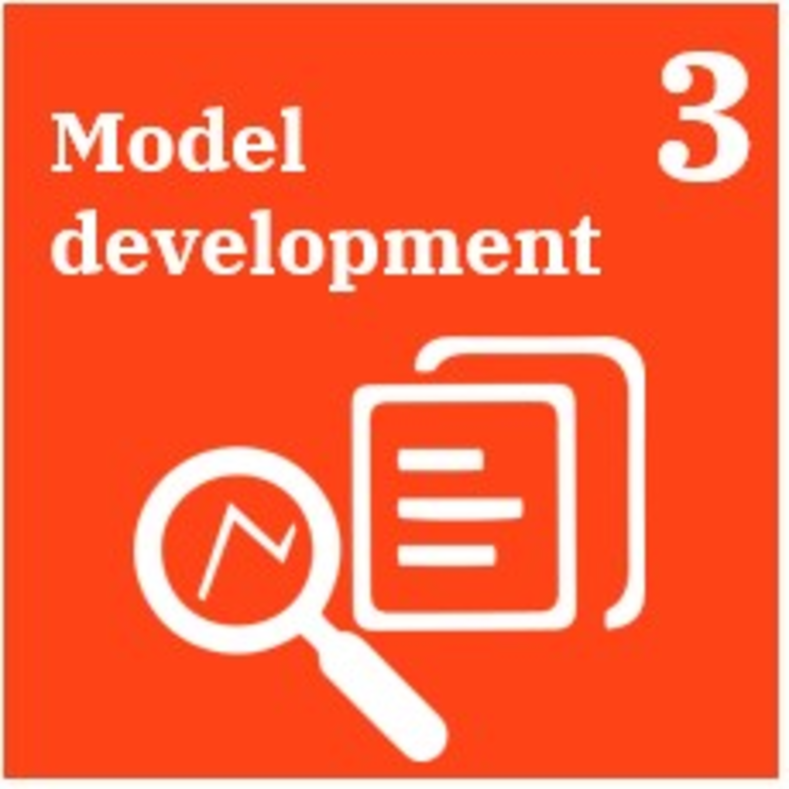

Supplement: S3 Fig — (TIF) [file pone.0300531.s003.tif]

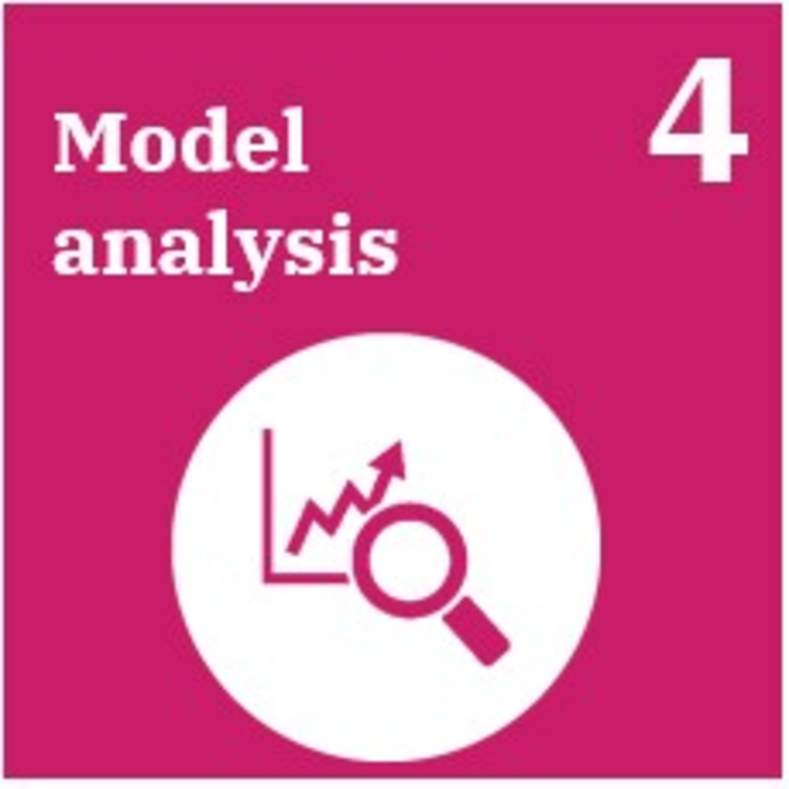

Supplement: S4 Fig — (TIF) [file pone.0300531.s004.tif]

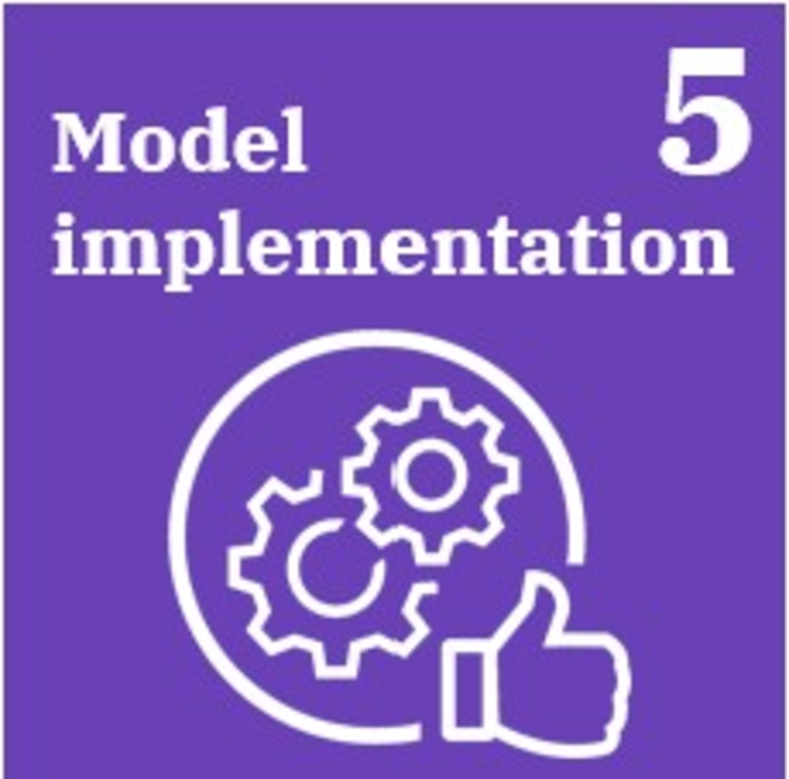

Supplement: S5 Fig — (TIF) [file pone.0300531.s005.tif]

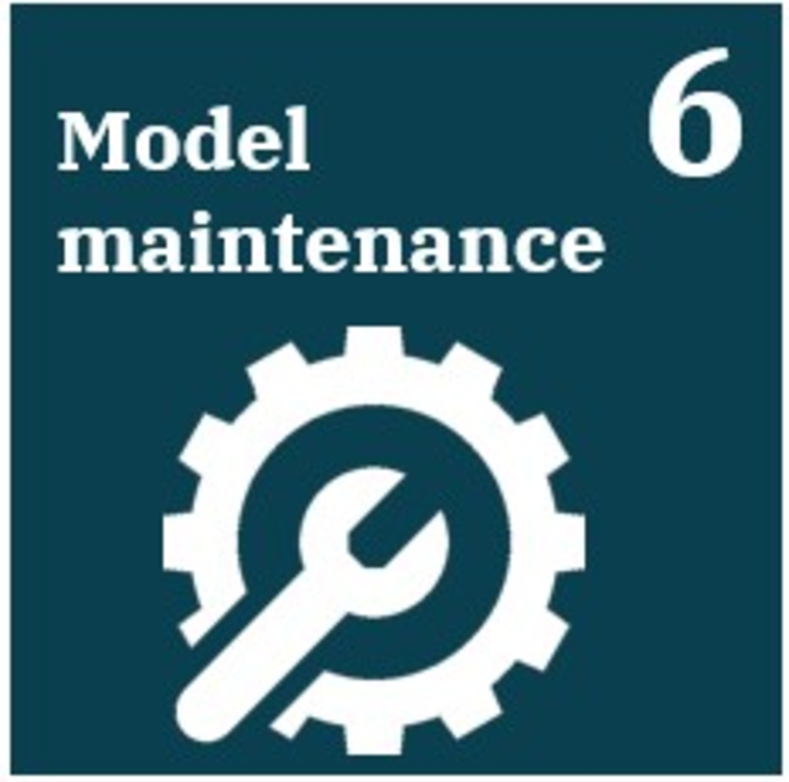

Supplement: S6 Fig — (TIF) [file pone.0300531.s006.tif]
